# Impact of Controlled Magneto‐Structural Coupling in MnCoGe‐Based Compounds on the Design of Multifunctional Materials for Technological Advances

**DOI:** 10.1002/advs.202508438

**Published:** 2025-07-29

**Authors:** Kang Liu, Xiaowen Hao, Wayne D. Hutchison, Stewart J. Campbell, Xiaoming Huang, Cuiping Zhang, Guoliang Li, Xuefei Miao, Fengjiao Qian, Jianli Wang, Qingyong Ren

**Affiliations:** ^1^ Department of Applied Physics Nanjing University of Aeronautics and Astronautics Nanjing 210016 China; ^2^ Institute of High Energy Physics Chinese Academy of Sciences Beijing 100049 China; ^3^ Spallation Neutron Source Science Center Dongguan 523803 China; ^4^ Guangdong Provincial Key Laboratory of Extreme Conditions Dongguan 523803 China; ^5^ School of Science UNSW Canberra at the Australian Defence Force Academy Australian Capital Territory 2600 Australia; ^6^ Key Laboratory of Artificial Structures and Quantum Control School of Physics and Astronomy Shanghai Jiao Tong University Shanghai 200240 China; ^7^ Beijing Key Lab of Microstructure and Property of Advanced Materials College of Materials Science and Engineering Beijing University of Technology Beijing 100124 China; ^8^ School of Materials Science and Engineering Nanjing University of Science and Technology Nanjing 210094 China; ^9^ Institute for Superconductivity and Electronic Materials University of Wollongong Wollongong 2500 Australia; ^10^ Center for Neutron Scattering and Advanced Light Sources Dongguan University of Technology Dongguan 523000 China

**Keywords:** lattice geometric compatibility, magnetocaloric effect, magneto‐structural coupling, MnCoGe, negative thermal expansion

## Abstract

Tailoring the magneto‐structural coupling in magnetic martensitic materials is pivotal for optimizing multifunctional properties such as magnetocaloric effect (MCE) and negative thermal expansion (NTE). This study demonstrates how Ni substitution in Mn_1‐_ *_x_* Ni*_x_* CoGe (*x* = 0.03 to 0.07) modulates the magneto‐structural transitions as investigated by *in‐situ* X‐ray diffraction, magnetization measurements, and geometric compatibility analysis. Ni doping is shown to stabilize the hexagonal phase, lower the martensitic transformation temperature, and introduce intermediate ferromagnetic hexagonal (FM‐Hex) states, thereby altering the transition pathways from a single paramagnetic hexagonal (PM‐Hex) ↔ ferromagnetic orthorhombic (FM‐Orth), to a two‐step PM‐Hex ↔ FM‐Hex ↔ FM‐Orth sequence. This modification in the magneto‐structural coupling alleviates lattice incompatibility, broadens phase transition temperature window, and enhances magnetization changes during the phase transition. The magnetocaloric refrigeration capacity increases from 213(14) J kg^−1^ for *x* = 0.04 to 308(18) J kg^−1^ for *x* = 0.07 under a 5 T driving field, while the NTE coefficient is tuned from ‐375(1) × 10^−3^ K^−1^ for *x* = 0.05 to ‐143(1) × 10^−3^ K^−1^ for *x* = 0.07. These findings provide mechanistic insights into the interplay between magnetization states and lattice compatibility, thereby advancing the design of energy‐efficient solid‐state cooling and precision actuators through controllable magneto‐structural coupling.

## Graphical abstract

In this work, the authors systematically investigate how Ni substitution regulates the magneto‐structural coupling and their functional consequences in the MnCoGe‐based compounds. Geometric nonlinear theory analysis reveals that Ni modification change of magneto‐structural transition can alleviate the lattice incompatibility, thereby broadening the operational temperature window of magnetocaloric effect (MCE) and reducing the negative thermal expansion (NTE) coefficient by flattening the magneto‐structural transitions.

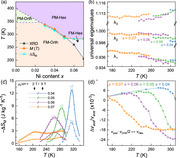

## 1 Introduction

Magnetic martensitic materials are gaining significant attention due to their ability to exhibit tunable magnetic properties under external stimuli such as magnetic fields, stress, and temperature.^[^ ^1^, ^2^ ^]^ These materials often display magneto‐structural coupling, where concurrent magnetic and structural phase transitions enable multifunctional behaviors, including shape memory effect,^[^ ^3^ ^]^ negative thermal expansion effect (NTE),^[^ ^4^, ^5^ ^]^ magnetocaloric effect (MCE),^[^ ^6^, ^7^, ^8^ ^]^ elastocaloric effect,^[^ ^9^, ^10^ ^]^ barocaloric effect (BCE)^[^ ^11^ ^]^ and giant magnetoresistance effect.^[^ ^12^ ^]^ Understanding the intrinsic mechanisms of such coupling is critical for optimizing performance in applications ranging from solid‐state refrigeration to precision actuators.^[^ ^13^, ^14^, ^15^, ^16^, ^17^ ^]^

Among magneto‐structural coupling materials, hexagonal MM'X compounds (M, M' = 3*d* transition element, X = Si, Ge) have emerged as a model system due to their tunable transition between high‐temperature hexagonal (austenite) and low‐temperature orthorhombic (martensite) phases.^[^ ^1^, ^18^, ^19^ ^]^ For instance, MnCoGe‐based compounds exhibit rich magneto‐structural evolution, where chemical doping or physical processing can tailor transition temperature and magnetization states.^[^ ^20^, ^21^, ^22^, ^23^, ^24^ ^]^ Such modifications have unlocked giant MCE,^[^ ^25^, ^26^ ^]^ BCE,^[^ ^27^, ^28^ ^]^ magnetostrictive,^[^ ^29^ ^]^ and NTE.^[^ ^30^ ^]^ Recent studies, such as those by Li and co‐workers^[^ ^19^, ^28^, ^31^, ^32^, ^33^ ^]^ and Guo and co‐workers,^[^ ^30^, ^34^, ^35^ ^]^ further demonstrate that synergistic compositional and microstructural design can enhance the MCE and NTE performances. Nonetheless, designing and optimizing magnetic martensitic materials remains challenging.

In MnCoGe‐based systems, the primary strategy involves regulating the martensitic transformation temperature between the Curie temperatures of the hexagonal phase (≈275 K) and the orthorhombic phase (≈345 K).^[^ ^1^, ^36^, ^37^, ^38^ ^]^ While this alignment aims to couple lattice and magnetic entropy changes, thereby maximizing the total entropy change during the phase transition, this approach introduces critical trade‐offs. First of all, first‐order magneto‐structural transitions often exhibit large hysteresis and reduced energy efficiency. Secondly, the phase transition typically occurs within a narrow temperature range, limiting practical applicability in MCE or NTE devices.^[^ ^39^, ^40^, ^41^, ^42^ ^]^ These challenges stem from an incomplete understanding of how magnetic states influence structural phase stability and lattice dynamics. For example, while structural transitions dominate functional behaviors like NTE, the role of magnetic order evolution in modulating transition energetics is often overlooked.^[^ ^43^, ^44^ ^]^ Addressing these gaps requires precise control over magneto‐structural couplings behaviors.

In this work, we have systematically investigated how Ni substitution in Mn_1‐_ *_x_* Ni*_x_* CoGe (*x* = 0.03‐0.07) regulates the magneto‐structural transitions and their functional consequences in the MnCoGe‐based compounds. By stabilizing the hexagonal structure and modifying the magneto‐structural coupling pathways, Ni doping relieves geometric incompatibility during the phase transition, broadens the operational temperature window, and reduces hysteresis losses. As a result, these synergistic effects enable concurrent optimization of the MCE and NTE, achieving high refrigeration capacities and tunable thermal expansion coefficients.

## 2 Results and Discussions

### 2.1 Phase Transition and Crystal Structures

**Figure 1:**
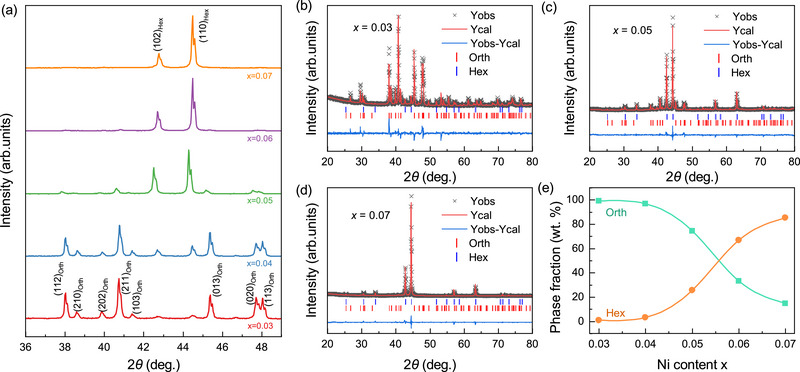
Crystal structure analysis of the Mn_1‐_ *_x_* Ni*_x_* CoGe (*x* = 0.03, 0.04, 0.05, 0.06, and 0.07) compounds. a) Room‐temperature XRD patterns. Miller indices for the main Bragg peaks are labeled. b‐d) Rietveld refinement of the XRD patterns for b) *x* = 0.03, c) *x* = 0.05, and d) *x* = 0.07. e) The mass‐weighted (wt) phase fractions of the hexagonal (Hex) and orthorhombic (Orth) phases as a function of Ni content.

The crystal structures of the Mn_1‐_ *_x_* Ni*_x_* CoGe (*x* = 0.03, 0.04, 0.05, 0.06, and 0.07) compounds were characterized via XRD at room temperature, as shown in **Figure** **1**a. Rietveld refinement of the XRD patterns^[^ ^45^ ^]^ reveals that all compounds adopt the Ni_2_In‐type hexagonal structure (space group: *P*6_3_/*mmc*, No. 194) or the TiNiSi‐type orthorhombic structure (*Pnma*, No. 62) or a mixture of these two structures, with no detectable impurity phases. Furthermore, the mass fractions of the hexagonal and orthorhombic phases at room temperature were extracted from the results (Figure 1b–e). The Miller indices of some of the prominent Bragg peaks are labeled in Figure 1a for both phases.

The Mn_0.97_Ni_0.03_CoGe sample predominantly exhibits the orthorhombic phase, with a minor presence of the hexagonal phase. As the Ni content increases, the hexagonal phase grows progressively (Figure 1e), eventually dominating the compounds. For instance, Mn_0.96_Ni_0.04_CoGe shows coexistence of the orthorhombic and hexagonal phases, while Mn_0.93_Ni_0.07_CoGe transitions to a primarily hexagonal phase. This systematic phase evolution implies that partial substitution of Mn with Ni stabilizes the hexagonal structure and lowers the martensitic transformation temperature.

### 2.2 Magnetization Properties

**Figure 2:**
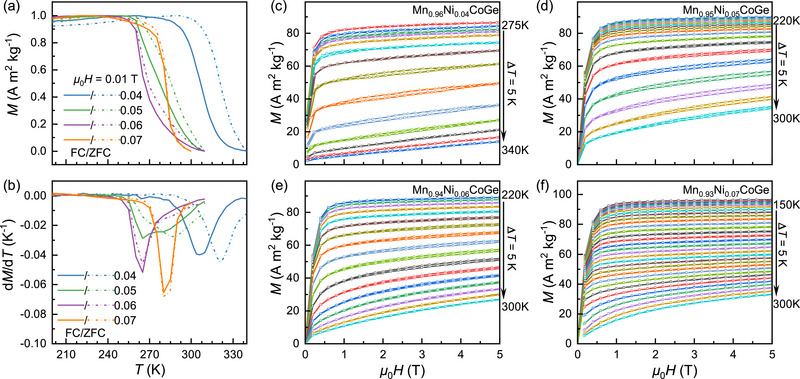
Magnetic properties of Mn_1‐_ *_x_* Ni*_x_* CoGe. a) Normalized temperature‐dependent magnetization, *M*(*T*), under 0.01 T, measured on warming after zero‐field cooling (ZFC, dashed lines) and field cooling (FC, solid lines). b) Temperature derivatives of magnetization (d*M*/d*T*) to determine magnetic transition temperature (*T*_C_). c–f) Isothermal magnetization curves, *M*(*μ*_0_ *H*), for *x* = 0.04, 0.05, 0.06, and 0.07 over the range of temperatures indicated.

The temperature‐dependent magnetization curves, *M*(*T*), for the Mn_1‐_ *_x_* Ni*_x_* CoGe compounds (*x* = 0.04, 0.05, 0.06, and 0.07) were measured under a small magnetic field of 0.01 T, with zero‐field cooling (ZFC, dashed lines) followed by field cooling (FC, solid lines), as shown in **Figure** **2**a. These compounds exhibit an abrupt magnetic transition from a low‐temperature ferromagnetic (FM) phase to a high‐temperature paramagnetic (PM) phase upon heating. The magnetic transition temperatures (*T*_C_) were determined by analyzing the derivative of the magnetization curve with respect to temperature (d*M*/d*T*), as illustrated in Figure 2b. For Mn_0.96_Ni_0.04_CoGe, *T*_C_ is identified as 320(3) K during heating and 305(3) K during cooling, displaying a clear thermal hysteresis and first‐order nature of the magnetic transition. Increasing *x* to 0.05 or 0.06 results in broader and even two‐step magnetic phase transitions, with *T*_C_ decreasing progressively to 264(3) K for *x* = 0.06. However, further increasing *x* to 0.07 shifts *T*_C_ back to the higher value of 280(3) K, accompanied by the complete disappearance of thermal hysteresis.

The isothermal magnetizations, *M*(*μ*_0_ *H*), for Mn_1‐_ *_x_* Ni*_x_* CoGe were measured across their respective magnetic phase transition temperature ranges. For Mn_0.96_Ni_0.04_CoGe (Figure 2c), the magnetization increases rapidly with applied magnetic field near and below 275 K, reaching saturation at ≈0.5 T, indicative of a FM state. As the temperature rises, the magnetization decreases gradually, while a pronounced magnetic hysteresis emerges, signaling a magnetic‐field‐driven metamagnetic transition. Above ≈315 K, the magnetization exhibits a linear dependence on the external field, the hysteresis vanishes, and the sample transitions to a paramagnetic state.

Similar behavior is observed in the compounds with *x* = 0.05 (Figure 2d) and 0.06 (Figure 2e), although the decrease in magnetization becomes more gradual and the magnetic hysteresis diminishes. Increasing the Ni dopant content to *x* = 0.07 further slows down the magnetization evolution rate with temperature, and the magnetic hysteresis disappears entirely (Figure 2f). These significant changes in the isothermal and isofield magnetization curves highlight a strong influence of the Ni doping concentration on the magnetic phase transition behavior.

### 2.3 Magnetocaloric Effect and Refrigeration Capacity

The MCE properties for the Mn_1‐_ *_x_* Ni*_x_* CoGe (*x* = 0.04‐0.07) compounds were evaluated by deriving the magnetocaloric entropy changes (‐Δ*S*_M_) from the experimental magnetization data using the Maxwell relation^[^ ^35^ ^]^:

(1) $$\Delta S\;\left(T,\mu_{0}\Delta H\right)=\mu_{0}\int_{0}^{\mu_{0}H}{\left(\frac{\partial M}{\partial T}\right)}_{\mu_{0}H}dH$$

**Figure 3:**
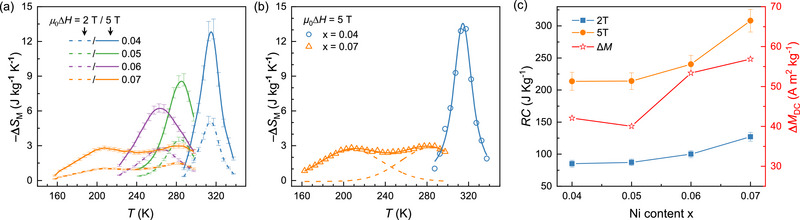
Magnetocaloric effects in Mn_1‐_ *_x_* Ni*_x_* CoGe. a) Magnetocaloric entropy changes (‐Δ*S*_M_) under *μ*_0_Δ*H* = 2 T and *μ*_0_Δ*H* = 5 T. b) Voigt function fitting (dashed lines) to ‐Δ*S*_M_ peaks for *x* = 0.04 (open circles) and *x* = 0.07 (open triangles) under *μ*_0_Δ*H* = 5 T. c) Refrigeration capacity (*RC*) at *μ*_0_Δ*H* = 2 T and *μ*_0_Δ*H* = 5 T and the magnetization difference (Δ*M*_DC_) between *T*_cold_ and *T*_hot_ (FWHM of ‐Δ*S*_M_ peaks) for all of the Mn_1‐_ *_x_* Ni*_x_* CoGe samples at *μ*_0_Δ*H* = 5 T.

**Figure** **3**a shows the ‐Δ*S*_M_ curves as a function of temperature under the magnetic field changes (μ_0_Δ*H*) of 2 T and 5 T. For *x* = 0.04, 0.05, and 0.06, a single prominent ‐Δ*S*_M_ peak is observed near the PM‐to‐FM magnetic transition temperature, as exemplified by the *x* = 0.04 compound in Figure 3b. The maximum entropy change ($-\Delta S_{M}^{\max}$) under a 5 T driving field are 13.1(0.9) J kg^−1^ K^−1^, 8.7(0.5) J kg^−1^ K^−1^, and 6.3(0.4) J kg^−1^ K^−1^ for *x* = 0.04, 0.05, and 0.06, respectively. The peak positions of the –Δ*S*_M_ values shift to lower temperatures in alignment with the *T*_C_ values indicated in Figure 2a,b, while the widths of the –Δ*S*_M_ peaks gradually broaden. It is notable that the *x* = 0.07 compound exhibits two distinct ‐Δ*S*_M_ peaks at 205(3) K and 280(3) K (Figure 3b), with maximum values of 2.8(0.2) J kg^−1^ K^−1^ and 3.0(0.2) J kg^−1^ K^−1^, respectively. The uncertainties in Figure 3a,c were estimated using the method in ref.[46] (see Section S1 in the Supporting Information).

The refrigeration capacity (*RC*)—a critical parameter for assessing MCE performance—is calculated from the ‐Δ*S*_M_ curves using the formula:

(2) $$\mathrm{RC}=\underset{T_{\mathrm{cold}}}{\int^{T_{\mathrm{hot}}}}\;{\left(\Delta S_{M}\right)}_{\mu_{0}H}\;dT$$

where *T*_cold_ and *T*_hot_ define the temperatures corresponding to the full width at half maximum (FHWM) of the entropy change peak. As shown in Figure 3c, *RC* increases from 85(5) J kg^−1^ and 214(14) J kg^−1^ for *x* = 0.04 to 127(7) J kg^−1^ and 308(18) J kg^−1^ for *x* = 0.07 under 2 T and 5 T driving fields, with this behavior different from the decreasing values of $-\Delta S_{M}^{\max}$ with increasing Ni content as noted above (Figure 3a). The magnetization difference (∆*M*) between *T*_cold_ and *T*_hot_, derived from the *μ*_0_Δ*H* = 5 T data of Figure 3c–f, also increases with increased Ni doping as shown in Figure 3c. This trend confirms a direct correlation between enhanced magnetization and improved refrigeration capacity, revealing the important role of Ni doping in optimizing the MCE performance of Mn_1‐_ *_x_* Ni*_x_* CoGe compounds. A comparison of the Δ*S*_M_ and *RC* data with those of similar magnetocaloric compounds reported in the past five years are summarized in Table S1 (Supporting Information). The materials in the present work rank among those with the highest values reported, showcasing superior magnetocaloric performance relative to state‐of‐the‐art counterparts.

Furthermore, the temperature averaged entropy change (*TEC*(Δ*T*_H − C_)), a figure of merit, is evaluated using the following formula^[^ ^7^ ^]^:

(3) $$\mathrm{TEC}\;\left(\Delta T_{H-C}\right)=\frac{1}{\Delta T_{H-C}}\max\underset{\Delta T\mathrm{mid}-\frac{\Delta T_{H-C}}{2}}{\int^{\Delta T\mathrm{mid}+\frac{\Delta T_{H-C}}{2}}}\left|\Delta S{\left(T\right)}_{\Delta H,T}\right|\mathrm{dT}$$

where Δ*T*_H − *C*_ is the temperature difference between hot and cold exchanges. The temperature averaged entropy change with *μ*_0_Δ*H* = 5 T are estimated as 10.7(0.7), 7.1(0.4), 5.3(0.3), and 2.6(0.2) J Kg^−1^ K^−1^ for *x* = 0.04, 0.05, 0.06, 0.07, respectively. In addition, the *TEC* for the same set of Ni doped materials with *μ*_0_Δ*H* = 2 T are 4.3(0.3), 2.9(0.2), 2.2(0.1), and 1.1(0.1) J Kg^−1^ K^−1^, respectively.

### 2.4 *In‐Situ* Analysis of Structural Phase Transitions

**Figure 4:**
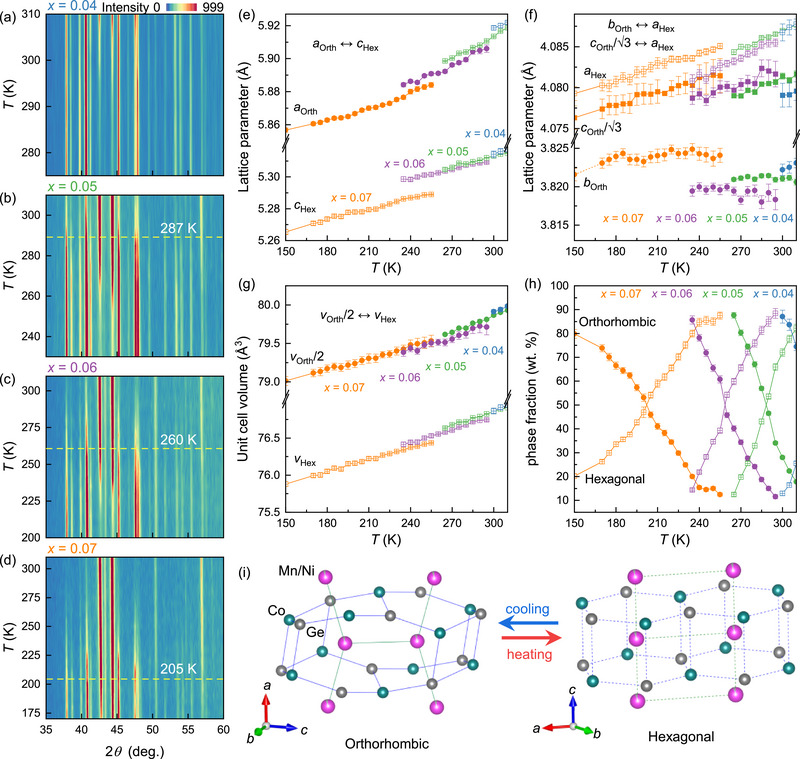
Structural and phase evolution during martensitic transformation of the Mn_1‐_ *_x_* Ni*_x_* CoGe compounds. a–d) *In‐situ* XRD patterns of Mn_1‐_ *_x_* Ni*_x_* CoGe for *x* = 0.4, 0.5, 0.6, 0.7 across phase transitions respectively with the martensitic transformation temperatures, *T*_t_, indicated for *x* = 0.5, 0.6, 0.7. e–g) Temperature‐dependent lattice parameters and unit cell volumes respectively. h) Temperature‐dependent phase fractions of the orthorhombic and hexagonal structures. i) Schematic forward/reverse martensitic transformation during cooling/heating.

In order to elucidate the relationship between the metamagnetic transition and the magnetocaloric effect, we performed in‐depth measurements across a wide temperature range to analyze the crystal structural evolution of the Mn_1‐_ *_x_* Ni*_x_* CoGe compounds. XRD thermal contour plots for *x* = 0.04, 0.05, 0.06, and 0.07 during heating are presented in **Figure** **4**a–d, respectively. As discussed in Section 3.1 (see also Figure 1e), at low temperatures the compounds adopt the orthorhombic TiNiSi‐type structure, while as the temperature increases, the diffraction peaks of the hexagonal Ni_2_In‐type structure intensify, signaling a martensitic transformation from the orthorhombic (martensitic) phase to the hexagonal (austenitic) phase.

The temperature‐dependent lattice parameters and unit cell volumes, derived from Rietveld refinements of the diffraction patterns, are summarized in Figure 4e–g. During the martensitic transformation, adjacent layers in the hexagonal structure undergo displacive shifts along the $\left\langle 1\bar{1}0\right\rangle$ direction, inducing pronounced anisotropic lattice distortions as demonstrated in Figure 4i. For instance, as evident from Figure 4e,f, the Mn_0.95_Ni_0.05_CoGe sample exhibits a sudden 11.25% contraction along the *a*_Orth_‐axis (transitioning to *c*_Hex_), a minor ≈0.14% expansion along *c*_Orth_/$\sqrt{3}$ (transitioning to *a*_Hex_), and a large ≈6.48% elongation along *b*_Orth_ (transitioning to *a*_Hex_). Notably, as in Figure 4g, the unit cell volume decreases by ≈3.90%, highlighting a substantial NTE during the phase transformation.

In Figure 4h we quantify the evolution of the orthorhombic and hexagonal phase fractions, highlighting the suppression of the orthorhombic phase with heating. The martensitic transformation temperature (*T*_t_), defined as the temperature at which 50% of the sample adopts the hexagonal phase, decreases systematically with increasing Ni content from *T*_t_ ≈ 287(3) K for *x* = 0.05 to *T*_t_ ≈ 205(2) K for *x* = 0.07 (Figure 4b–d); this behavior mirrors the shifting of the ‐Δ*S*_M_ peak in the MCE measurements (Figure 3), inferring simultaneous crystal structural and magnetic phase transitions.

### 2.5 Negative Thermal Expansion

**Figure 5:**
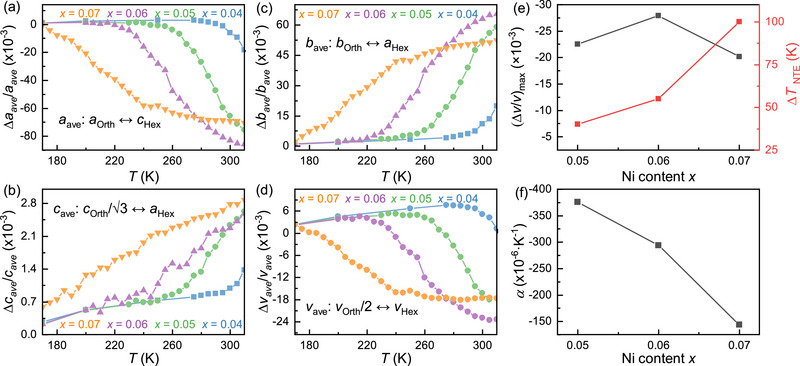
Thermal expansion behavior of Mn_1‐_ *_x_* Ni*_x_* CoGe. a–d) Temperature‐dependent average lattice parameters (*a*_ave_, *b*_ave_, *c*_ave_) and unit‐cell volumes (*V*_ave_) respectively. e) Maximum relative volume change (Δ*V/V*) and transformation temperature window (Δ*T*_NTE_, corresponding to orthorhombic phase fraction: 15% to 85%) for *x* = 0.05, 0.06, and 0.07. f) Linear thermal expansion coefficients (*α*) for *x* = 0.05, 0.06, and 0.07 (see Figure S1, Supporting Information, for the data of *x* = 0.04).

As revealed by the *in‐situ* XRD, the structural transformation from the orthorhombic phase to the hexagonal phase involves significant lattice distortion and NTE. To quantify this NTE phenomenon, we define the average lattice parameters as *L*_ave_ = *L*_Orth_× *f*_Orth_ + *L*_Hex_× *f*_Hex_, where *f*_Orth_ and *f*_Hex_ are the fractions of the orthorhombic and hexagonal phases.^[^ ^22^, ^30^ ^]^ The thermal expansion across the martensitic transformation is calculated using (*L*_ave_‐*L*_0_)/*L*_0_, where *L*_0_ denotes the reference lattice parameters below the transition temperature window. As shown in **Figure** **5**a–d, the *a*_ave_‐axis contracts dramatically during heating, while the *b*_ave_‐ and *c*_ave_‐ axes expand slightly, resulting in an overall negative thermal expansion in the unit cell volume (*V*_ave_).

Although the Mn_1‐_ *_x_* Ni*_x_* CoGe (*x* = 0.04, 0.05, 0.06, and 0.07) compounds exhibit similar NTE trends during the martensitic transformation, their temperature‐dependent evolution rates differ significantly, becoming less pronounced with increasing Ni content. Notably, the *x* = 0.07 compound displays a flatter thermal expansion profile. The maximum relative unit cell volume change (Δ*V*/*V*)_max_ and the transformation temperature window (Δ*T*_NTE_), as defined by the fractional range of the orthorhombic phase from 15% to 85% are compared in Figure 5e. While (Δ*V*/*V*)_max_ remains relatively consistent (≈20 × 10^−3^ to ≈28 × 10^−3^) across *x* = 0.05, 0.06, and 0.07, Δ*T*_NTE_ increases monotonically with increasing Ni content (Figure 5e). This broadening aligns with the widening magnetic entropy (Δ*S*_M_) curves in Figure 3. The linear thermal expansion coefficients (*α*) are determined as ‐376(1) × 10^−6^, ‐294(1) × 10^−6^, and ‐144(1) × 10^−6^ K^−1^ for *x* = 0.05, 0.06, and 0.07, respectively, underscoring the role of Ni doping in moderating the negative thermal expansion behavior.

It is noted that the NTE in MnCoGe‐based alloys is fully repeatable as the first‐order magneto‐structural transition is inherently reversible. Although they suffer severe mechanical failure due to the strong lattice distortion during the phase transition, compounding them with low‐melting metals^[^ ^39^ ^]^ and epoxy^[^ ^30^ ^]^ or introducing grain texture^[^ ^19^, ^35^ ^]^ can enhance the mechanical performance and ensure the potential for practical application of such materials.

### 2.6 Variation of Magneto‐Structural Coupling and Geometric Compatibility

**Figure 6:**
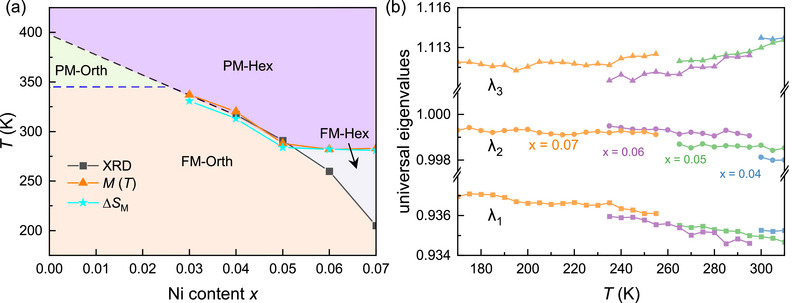
Magneto‐structural coupling and geometric compatibility analysis in Mn_1‐_ *_x_* Ni*_x_* CoGe compounds. a) Temperature‐composition phase diagram illustrating the transitions between the paramagnetic hexagonal (PM‐Hex), the ferromagnetic hexagonal (FM‐Hex), and the ferromagnetic orthorhombic (FM‐Orth) phases. b) The temperature‐dependent geometric compatibility eigenvalues between hexagonal and orthorhombic phases for *x* = 0.04 to *x* = 0.07. Eigenvalues that are closer to 1 indicate improved lattice compatibility.

According to the above results, Ni doping plays a critical role in modulating the magnetic and structural phase transitions and the behavior of the magnetocaloric effect and the negative thermal expansion in MnCoGe‐based compounds. In order to interrelate these phenomena, a comprehensive magneto‐structural phase diagram has been constructed in **Figure** **6**a based on the *in‐situ* XRD, magnetization, and magnetic entropy data.^[^ ^23^, ^47^ ^]^ At low Ni content, e.g. *x* = 0.04, the system undergoes a coupled magneto‐structural transition between the paramagnetic hexagonal (PM‐Hex) phase and the ferromagnetic orthorhombic (FM‐Orth) phase. As *x* increases, and as shown in Figure 4, the martensitic transformation temperature *T*_t_ decreases, altering the coupling behavior. For *x* = 0.06, the transition occurs via an intermediate ferromagnetic hexagonal (FM‐Hex) phase rather than a direct PM‐Hex ↔ FM‐Orth transformation. This intermediate step explains the two‐step magnetic phase transition observed in the d*M*/d*T* versus *T* curves (Figure 2b). At *x* = 0.07, *T*_t_ drops further, leading to a distinct two‐phase‐transitions sequence: PM‐Hex ↔ FM‐Hex ↔ FM‐Orth. This dual transition manifests as two peaks in the ‐Δ*S*_M_ curves (Figure 3b) and correlates with the enhanced magnetization differences Δ*M* (Figure 3c), ultimately increasing the refrigeration capacity, *RC*, with Ni doping.

Furthermore, the width of the phase transition temperature window Δ*T*_NTE_, is shown to be closely tied to lattice compatibility during the first‐order magnetic martensitic transformation. We analyzed the geometric compatibility using the 3 × 3 transformation stretch tensor^[^ ^23^, ^44^, ^48^ ^]^:

(4) $$U=\left[\begin{array}{ccc} \lambda_{1} & 0 & 0\\ 0 & \lambda_{2} & 0\\ 0 & 0 & \lambda_{3} \end{array}\right]=\left[\begin{array}{ccc} \frac{b_{\mathrm{Orth}}}{a_{\mathrm{Hex}}} & 0 & 0\\ 0 & \frac{c_{\mathrm{Orth}}}{\sqrt{3}a_{\mathrm{Hex}}} & 0\\ 0 & 0 & \frac{a_{\mathrm{Orth}}}{c_{\mathrm{Hex}}} \end{array}\right]\;$$

where the eigenvalues are derived from the lattice constants of the two phases. The closer the eigenvalue *λ*_2_ is to 1, the better the geometric compatibility of the phase transition between hexagonal and orthorhombic.

As shown in Figure 6b, *λ*_2_ for all samples approaches unity as the temperature decreases, indicating enhanced geometric compatibility between the orthorhombic and hexagonal phases. Furthermore, in the PM‐Hex ↔ FM‐Orth coupled systems, *λ*_2_ converges toward 1 with increasing Ni content (e.g., *x* = 0.04→0.05→0.06). However, this trend halts when the magneto‐structural coupling evolves into the PM‐Hex ↔ FM‐Hex ↔ FM‐Orth in *x* = 0.07. The overall improvement in the lattice matching with higher Ni doping and lower phase transition temperatures reduces the energy barriers caused by lattice incompatibility during the magneto‐structural transition. Consequently, the phase transition proceeds more gradually, eliminating the need for significant supercooling or superheating to drive the transformation within a narrow temperature range.

Finally, it is worth noting that, although Ni doping at *x* = 0.07 lowers the Curie temperature below room temperature and results in a broader Δ*S*_M_ peak, this behavior has several notable practical benefits:

1) The dual Δ*S*_M_ peaks of the *x* = 0.07 sample span a wide temperature range from ≈190 K to ≈300 K. This enables continuous magnetocaloric refrigeration bridging the deep‐freezing temperature region (e.g., from ≈200 K to ≈255 K^[^ ^9^ ^]^) and the room temperature regimes, thereby enhancing operational versatility.

2) The *x* = 0.07 sample exhibits significantly suppressed hysteresis effects. This reduction minimizes energy losses associated with magnetic irreversibility, thereby improving refrigeration efficiency and cyclic stability in practical devices.

3) The alleviated lattice incompatibility in *x* = 0.07 lowers the phase transition energy barrier. This broadens the magneto‐structural transition window and moderates the NTE coefficient, aligning the material's properties more closely with the demands of practical application.

## 3 Conclusion

In summary, this study demonstrates that Ni substitution in MnCoGe alloys provides a powerful lever to tune their magnetocaloric effect and negative thermal expansion properties by modulating magneto‐structural coupling pathways. Through systematic doping, we achieve a controlled shift from PM‐Hex ↔ FM‐Orth transition at lower Ni content (*x* ≤ 0.06) to a two‐step PM‐Hex ↔ FM‐Hex ↔ FM‐Orth transition at *x* = 0.07, thereby widening the operating temperature windows (Δ*T* > 50 K) and improving the magnetocaloric refrigeration capacities (*RC* > 300 J kg^−1^ under 5 T driving field in *x* = 0.07). Geometric compatibility analysis reveals that the change of magneto‐structural coupling can alleviate the lattice incompatibility and reduce energy barriers, thereby flattening the magneto‐structural transitions and reducing the NTE coefficient. These insights will contribute to resolving critical challenges in magnetic martensitic materials, such as hysteresis losses and narrow functional ranges, by linking atomic‐scale coupling mechanisms to macroscopic performance, thereby offering guidance for designing multifunctional materials with tailored MCE and NTE properties for energy‐efficient solid‐state cooling and precision actuator technologies.

## 4 Experimental Section

Mn_1‐_ *_x_* Ni*_x_* CoGe polycrystalline samples (*x* = 0.03, 0.04, 0.05, 0.06, 0.07) were synthesized via arc melting under an argon atmosphere (argon pressure ≈0.7 bar) using stoichiometric amounts of high‐purity Mn, Co, Ge, and Ni (> 99.95 wt%). In order to compensate for Mn evaporation during melting, 3 wt% excess Mn was added. The ingots were melted four times and flipped between melts as an aid to homogeneity. The resultant ingots were fully homogenized by heat treatment as follows: the ingots were wrapped in tantalum foil and sealed in quartz glass tubes with high vacuum, annealed at 1123 K for 5 days, and subsequently quenched into ice water.

Room temperature X‐ray diffraction (XRD) patterns were acquired using a Rigaku SmartLab diffractometer with Cu‐*K*_α_ (*λ* = 0.154 nm) radiation to verify phase purity. *In‐situ* temperature‐dependent XRD measurements (150 to 310 K) were performed on a PANalytical Empyrean diffractometer equipped with a cryostat. The measurements were carried out over the 2*θ* angular range 15° ≤ 2*θ* ≤ 110° with a scanning step of 0.02°. The resultant XRD patterns were analyzed using the Rietveld method with Fullprof software.^[^ ^45^ ^]^ The variations of crystal structures, lattice parameters, and phase fractions with temperature were derived from the refinement results.

The magnetic properties were characterized using a Quantum Design Physical Properties Measurement System (PPMS) over the temperature range ≈160 to 340 K. Temperature‐dependent iso‐field magnetization curves were measured under a magnetic field of 0.01 T during heating and cooling cycles (200–340 K). Isothermal magnetization loops (0 to 5 T) were collected at 5 K intervals across the phase transition range to evaluate the field‐induced responses.

## Conflict of Interest

The authors declare no conflict of interest.

## Author Contributions

K.L. and X.H. contributed equally to this work. K.L. and X.H. write original draft, X.H. performed investigate, funding acquisition, formal analysis, and data curation. W.D.H. and S.J.C. performed *in‐situ* X‐ray diffraction measurements. X.H. and C.Z. performed software analysis. G.L. and X.M. performed data curation. J.W. and F.Q. performed formal analysis and funding acquisition. Q.R. write review & editing, supervision, funding acquisition, conceptualization, investigation, writing—original draft, project administration, formal analysis, methodology, resources.

## Supporting information



Supporting Information

## Data Availability

The data that support the findings of this study are available from the corresponding author upon reasonable request.
