# Supplementary material for: Impact of Controlled Magneto‐Structural Coupling in MnCoGe‐Based Compounds on the Design of Multifunctional Materials for Technological Advances
Source: Adv Sci (Weinh). 2025 Jul 29;12(39):e08438. doi: 10.1002/advs.202508438 (PMC12533387; doi:10.1002/advs.202508438)
Supplement: Supplementary file 1 — Supporting Information [file ADVS-12-e08438-s001.docx]

Supporting Information

Impact of Controlled Magneto-Structural Coupling in MnCoGe-Based Compounds on the Design of Multifunctional Materials for Technological Advances

Kang Liu, Xiaowen Hao, Wayne D. Hutchison, Stewart J. Campbell, Xiaoming Huang, Cuiping Zhang, Guoliang Li, Xuefei Miao, Fengjiao Qian*, Jianli Wang*, Qingyong Ren *

The file includes:

Methods of estimation of uncertainties

Supplementary text

Tables S1

Figures S1

References

**1. Estimation of uncertainties**

The uncertainties in **Figure 3**a and c were estimated using the method in Ref. [1]:

$$\sigma\left| \Delta S_{M}\left( T_{\mathrm{av}} \right)_{\Delta H} \right|=\frac{1}{2\left| \delta T \right|}\left\{ \left| \delta H \right|\times\left( \sigma M_{1}+2\sum_{k=2}^{n-1} \sigma M_{k}+\sigma M_{n} \right)+\left( \left| \delta M_{1} \right|\sigma H_{1}+2\sum_{k=2}^{n-1} \left( \left| \delta M_{k} \right|\sigma H_{k} \right)+\left| \sigma M_{n} \right|\sigma H_{n} \right)+2\left| \Delta S_{M}\left( T_{\mathrm{av}} \right)_{\Delta H} \right|\times\left( \sigma T_{u}+\sigma T_{l} \right) \right\}$$

$T_{\mathrm{av}}= \left( T_{u}+T_{l} \right)/2$ is the average temperature from two isothermal magnetization values measured at $T_{u}$ and $T_{l}$, $\delta T = T_{u}-T_{l}$ is the temperature difference between the two isotherms, *n* is the number of points measured for each of the two isotherms, $\delta H = \Delta H/\left( n-1 \right)$, and $\delta M_{k}= \left[ {M\left( T_{u} \right)}_{k}+{M\left( T_{l} \right)}_{k} \right]$, and $\sigma M_{k}= \left[ \sigma{M\left( T_{u} \right)}_{k}+\sigma{M\left( T_{l} \right)}_{k} \right]$ is the sum of the errors in the magnetization measured at $T_{u}$ and $T_{l}$ for magnetic field $H_{k}$. $\sigma H_{k}= \left[ \sigma{H\left( T_{u} \right)}_{k}+\sigma{H\left( T_{l} \right)}_{k} \right]$ is the sum of the errors in the magnetic field $H_{k}$ at $T_{u}$ and $T_{l}$. Here, we assumed an accuracy of the magnetization measurements of $\sigma M_{k}= 0.02\% \times M_{k}$, an accuracy of the magnetic field of $\sigma H_{k} = 0.01\%\times H_{k}$ and the errors for temperature of $\sigma T = 0.05\% \times T$.

The uncertainties for the negative thermal expansion (NTE) were estimated directly from the Rietveld refinements.

**2. Comparison of magnetocaloric effects**

**Table S1.** Comparison of the magnetic entropy changes (Δ*S*_M_) and values of the refrigeration capacity (*RC*) of the present Ni-doped MnCoGe compounds with similar magnetocaloric compounds reported in the past five years.

| Sample | *T*_M_ (K) | -Δ*S*_M_  (J kg^-1^ K^-1^) | *RC*  (J kg^-1^) | Δ*H* (T) | Ref. |
| --- | --- | --- | --- | --- | --- |
| Mn_0.96_Ni_0.04_CoGe | **314** | **13.1(0.9)** | **214(14)** | **5** | **This work** |
| Mn_0.95_Ni_0.05_CoGe | **285** | **8.7(0.5)** | **214(13)** | **5** | **This work** |
| Mn_0.94_Ni_0.06_CoGe | **264** | **6.3(0.4)** | **240(14)** | **5** | **This work** |
| Mn_0.93_Ni_0.07_CoGe | **205 (*T*_M_)/**  **280 (*T*_C_)** | **2.8(0.2)/**  **3.0(0.2)** | **308(18)** | **5** | **This work** |
| Mn_0.94_Fe_0.12_Co_0.94_Ge | 320 | 23.7 | 200.6 | 5 | ^[2]^ |
| Mn_0.93_Fe_0.14_Co_0.93_Ge | 286 | 22.1 | 213.7 | 5 | ^[2]^ |
| MnCoGe_0.946_Ga_0.054_ | 310 | 31.1 | 250 | 5 | ^[3]^ |
| MnCoGe_0.96_V_0.04_ | 317 | 3.32 | 178.44 | 3 | ^[4]^ |
| MnCo_0.94_Bi_0.06_Ge | 246 | 26.42 | 302.49 | 5 | ^[5]^ |
| Mn_0.97_CoGe | 265 | 27.6 | 212.11 | 5 | ^[6]^ |
| MnCoGeSi film | 270 | 2.5 | 89 | 5 | ^[7]^ |
| Mn_0.95_Cd_0.05_CoGe | 322 | 8.006 | 281.8 | 5 | ^[8]^ |
| MnCo_0.7_Fe_0.3_Ge_0.8_Si_0.2_ | 265 | 12.2 | 112.8 | 5 | ^[9]^ |
| MnCo_0.94_Gd_0.06_Ge | 279/334 | 2.6 | 282 | 5 | ^[10]^ |
| MnCoGe_0.97_Al_0.03_ | ~345 | 4.93 | - | 5 | ^[11]^ |
| Mn_0.9_Nb_0.1_CoGe | 275/325 | 3.30/2.13 | 307 | 5 | ^[12]^ |
| Mn_0.89_Cu_0.11_CoGe | 281 | 58 | 258.2 | 5 | ^[13]^ |
| Mn_0.94_Ag_0.06_CoGe | 270 | 22 | 308 | 5 | ^[14]^ |

**3. Negative thermal expansions**

The shaded regions of **Figure S1** indicate the uncertainty associated with the inability to measure the lattice parameters for *x* = 0.04 over the entire temperature window of the phase transition (see e.g. **Figure 5**a). This is due to the limited temperature range of ~150 - 310 K available to the sample environment of our *in-situ* X-ray diffractometer. The values for *x* = 0.04 in **Figure S1** are estimated based on the limited data available.

**Figure S1.** a) Maximum relative volume change (Δ*V/V*)_max_ and transformation temperature window (Δ*T*_NTE_, corresponding to orthorhombic phase fraction: 15% to 85%) for *x* = 0.04, 0.05, 0.06, and 0.07. b) Linear thermal expansion coefficients (*α*) for *x* = 0.04, 0.05, 0.06, and 0.07. Note: the dashed lines indicate that the values for x = 0.04 are estimated based on the limited data available.

**References:**

[1] V.K. Pecharsky, K.A. Gschneidner, Magnetocaloric effect from indirect measurements: Magnetization and heat capacity, Journal of Applied Physics, 86 (1999) 565-575.

[2] X.W. Hao, Y. Zhao, X.M. Huang, K. Liu, B. Zhao, C. Zhang, J. Ma, F. Qian, X. Miao, Y. Kuang, H. Yan, Z. Li, L. Zuo, X. Tong, B. Yang, Q. Ren, *In-situ* study of the temperature effects on the magneto-structural transition in the MnCoGe-based magnetocaloric compounds, Acta Materialia, 296 (2025) 121241.

[3] R.G. Suárez, I. Betancourt, M. López-Cruz, J. Zamora, J.L.S. Llamazares, J. Matutes-Aquino, Enhanced magnetocaloric effect in MnCoGe alloys with gallium additions, Journal of Alloys and Compounds, 1018 (2025) 179170.

[4] X. Wang, H. Zhang, J. Shi, X. Zhang, M. Yue, Tuning structural transformations in MnCoGe system: The role of vanadium-induced d-d hybridization, Journal of Alloys and Compounds, 1010 (2025) 177849.

[5] X. Sun, Y. Huang, J. Wu, Y. Wu, T. Wu, S. Zhao, Tuning martensitic transformation and magnetocaloric effect with Bi substitution in MnCo_1-x_Bi_x_Ge alloys, Journal of Magnetism and Magnetic Materials, 590 (2024) 171663.

[6] R. Li, L. Wang, P. Miao, C. Xie, X. Tang, G. Tan, Fine tuning of Mn/Co vacancies for optimized magnetocaloric performance in MnCoGe alloys, Journal of Magnetism and Magnetic Materials, 603 (2024) 172224.

[7] Y. Xiao, F. Qian, X. Gao, R. Zhao, X. Miao, H. Yang, Structural, magnetic and magnetocaloric properties of hexagonal MnCoGe-based thin films, Ceramics International, 49 (2023) 18180-18186.

[8] X. Si, R. Zhang, J. Tan, B. Ge, Y. Liu, Evolution of magnetic and magnetocaloric behavior in Mn_1-x_Cd_x_CoGe intermetallics, Journal of Alloys and Compounds, 968 (2023) 171714.

[9] Y. Li, X. Ye, L. Li, E. Liu, Emergent evolution of first-order phase transitions from magneto-structural to magneto-elastic in MnCo_1−y_Fe_y_Ge_1−x_Si_x_ alloys, Journal of Physics: Energy, 5 (2023) 034008.

[10] Y. Li, L. Qin, X. Zhang, Achievement of reversible table-like magnetocaloric effect in MnCo_1-x_Gd_x_Ge alloys around room temperature, Materials Letters, 326 (2022) 132975.

[11] A.R.A. Rahman, M.F. Md Din, N.K. Othman, J. Wang, N.S. Suhaimi, S.X. Dou, N.F. Mohamed Sharif, N.F. Makmor, The Critical Behaviour and Magnetism of MnCoGe_0.97_Al_0.03_ Compounds, Crystals, 12 (2022) 205.

[12] K. Kutynia, P. Gębara, A. Przybył, The Structure and Magnetocaloric Effect of MnCoGe Alloy Modified by Nb, Archives of Metallurgy and Materials, (2021) 879-883.

[13] S.K. Pal, C. Frommen, S. Kumar, B.C. Hauback, H. Fjellvåg, G. Helgesen, Enhancing giant magnetocaloric effect near room temperature by inducing magnetostructural coupling in Cu-doped MnCoGe, Materials & Design, 195 (2020) 109036.

[14] A. Aryal, S. Pandey, I. Dubenko, D. Mazumdar, S. Stadler, N. Ali, Magnetostructural phase transitions and large magnetic entropy changes in Ag-doped Mn_1−x_Ag_x_CoGe intermetallic compounds, MRS Communications, 9 (2019) 315-320.
